# Histopathologic Evaluation of Atypical Fibroxanthoma or Pleomorphic Dermal Sarcoma Debulk Specimen from Mohs Surgery: A Requirement for Their Proper Distinction

**DOI:** 10.3390/dermatopathology11030019

**Published:** 2024-07-03

**Authors:** Muhammad N. Mahmood

**Affiliations:** Department of Laboratory Medicine and Pathology, University of Alberta Hospital, Edmonton, AB T6G 2B7, Canada; mmahmood@ualberta.ca; Tel.: +1-780-407-2145

**Keywords:** atypical fibroxanthoma, pleomorphic dermal sarcoma, debulk specimen, dermatologic surgery, Mohs

## Abstract

Pleomorphic dermal sarcomas can be clinically aggressive, with a higher tendency to cause local recurrence, metastasis, and death. Atypical fibroxanthoma and pleomorphic dermal sarcoma are histopathologically similar, and their distinction requires a systematic examination of the entire excised tumor. Since Mohs micrographic surgery is commonly utilized to treat atypical fibroxanthoma, a histopathologic evaluation of debulk specimens by permanent pathology is prudent to avoid underdiagnosing pleomorphic dermal sarcoma. This approach can improve risk assessment and treatment decisions, ultimately enhancing patient outcomes. Also, the proper distinction will facilitate the future development of accurate staging criteria and additional treatment modalities.

## 1. Introduction

Atypical fibroxanthoma (AFX) and pleomorphic dermal sarcoma (PDS) are two genetically linked ends of the same spectrum [1]. While AFX is an early-stage sarcoma, PDS is a more advanced and aggressive form, with a higher recurrence rate and greater propensity to metastasize [2]. Due to the substantial difference in their clinical behavior, it is critical to distinguish between AFX and PDS accurately. However, this distinction cannot be made by partial or superficial biopsies alone, as it requires a comprehensive histopathologic examination of the whole tumor. Since Mohs micrographic surgery (MMS) is often employed for these tumors, it is crucial to consider the histopathologic evaluation of the debulk specimen (DS) by permanent pathology to avoid overlooking PDS. This contribution discusses the distinction between these two tumors and underscores the importance of a histopathologic evaluation of the DS by a dermatopathologist to distinguish between them.

## 2. Distinction between AFX and PDS

**Figure 1:**
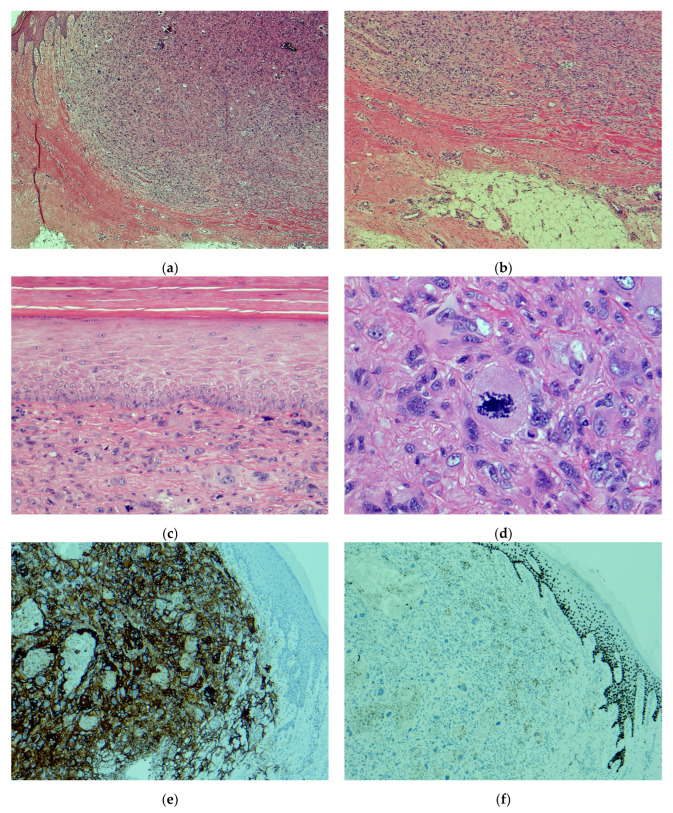

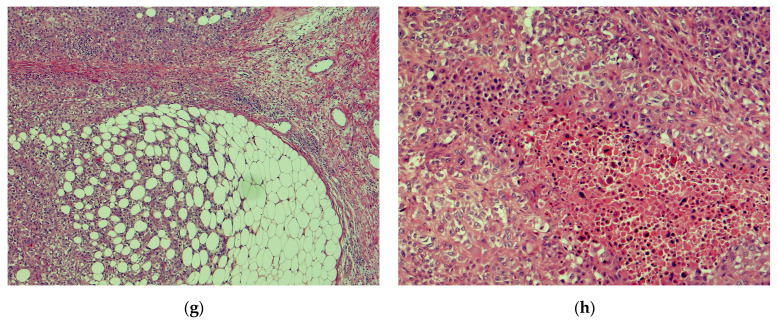
Microscopic features and the distinction between atypical fibroxanthoma and pleomorphic dermal sarcoma: (**a**) AFX characterized by a highly cellular dermal-based neoplasm (H&E, ×25); (**b**) AFX confined to the dermis with no microscopic infiltration of the underlying subcutis (H&E, ×50); (**c**) AFX formed by atypical epithelioid cells with nuclear pleomorphism and no evidence of connection or origin from surface epidermis (H&E, ×200); (**d**) AFX displaying striking nuclear pleomorphism and brisk mitotic activity with atypical forms (H&E, ×400); (**e**) although not a specific immunohistochemical marker, CD10 often shows positivity in AFX (CD10, ×50); (**f**) the p40 stain is negative in AFX, helping differentiate it from cutaneous squamous cell carcinoma (p40, ×50); (**g**) PDS showing a frank invasion of the subcutis, the main feature differentiating it from AFX (H&E, ×50); (**h**) PDS displaying tumor necrosis, a feature seen in up to 50% of cases (H&E, ×100).

Atypical fibroxanthoma is a dermal-based cutaneous tumor of undetermined differentiation; however, it is generally categorized within the mesenchymal neoplasms. They primarily arise on sun-exposed sites in the elderly, notably the head and neck [3]. When viewed under a microscope, they are usually characterized as dermal tumors, which are highly cellular, with nuclear pleomorphism and brisk mitotic activity (Figure 1a–d). A panel of immunohistochemical stains, mainly comprised of markers for keratinocytic, melanocytic, and mesenchymal tumors, is employed to exclude melanoma, poorly differentiated squamous cell carcinoma, and other sarcomas (Figure 1e,f). Despite their atypical histomorphology, when strict criteria are followed, most AFX behave in a low-grade fashion.

Efforts to differentiate AFX from PDS using cytological features, immunohistochemical markers, and molecular tools have yet to prove helpful in routine practice. AFX and PDS are two genetically related ends of the same spectrum, with the former representing the early stage and the latter representing the more advanced stage [1]. Tumors similar to AFX in terms of their clinical, histopathological, and immunohistochemical profiles, but with infiltration of the subcutis and deeper soft tissue, lymphovascular and perineural invasion, or necrosis, are known as PDS [2] (Figure 1g,h). Tumors with even minimal or focal subcutaneous infiltration have shown adverse outcomes [4]. There is no specific size demarcation between AFX and PDS; however, PDSs usually tend to be bigger tumors, with a median size of 2.5 cm. 

Recent studies disclose similar molecular profiles and no significant genetic differences between AFX and PDS [1]. They are highly mutated tumors and exhibit a range of DNA copy number alterations. Frequent mutations involve *TP53*, *TERT promoter*, *FAT1*, *NOTCH1*, *NOTCH2*, and *CDKN2A*. Infrequent *RAS* mutations are seen in PDS and not in AFX; however, they are encountered in only 9% (3 of 35 cases) of PDSs. The genetic changes in AFX and PDS have also been observed in cutaneous squamous cell carcinomas. These tumors develop in regions of the skin that are frequently exposed to sunlight, suggesting that the commonality in their mutation profiles is caused, at least in part, by a shared pathogenesis induced by ultraviolet exposure.

Compared to AFX, PDS is more aggressive and has a higher local recurrence rate (range of 20–30%) and a greater risk of metastasis. PDS can spread to the skin (including satellite metastasis), lymph nodes, and lungs (metastatic rate range of 10–20%), and this can lead to a significant risk of mortality (up to 20%) [5]. Effective treatments for metastasized PDS are still lacking. The dynamic between AFX and PDS is perhaps comparable to that between low-risk superficial cutaneous squamous cell carcinoma (SCC) and high-risk cutaneous SCC that has poor differentiation, deep infiltration, or perineural invasion. As the current concept and terminology of PDS was presented in 2012, some practitioners may not be well-versed in its features and relationship with AFX [2]. Several tumors which were formerly classified as aggressive AFX would now be designated as PDS.

## 3. Shave Biopsies to Distinguish between AFX and PDS

The histopathologic evaluation of shave biopsies cannot distinguish between AFX and PDS. In a retrospective cohort analysis of 75 cases, nearly all initial biopsies that produced a diagnosis of AFX were superficial and only captured the papillary or upper reticular dermis [6]. The distinction between AFX and PDS in these initial biopsies was not possible until excision or recurrence allowed for the histopathologic evaluation of a larger specimen. In a separate study, 75% (12 of 16) of PDS cases were initially diagnosed as AFX and subsequently reclassified as PDS [7]. Reports of AFX treated with MMS recurring as PDS or being reclassified post-surgery as PDS have also been documented [8,9].

## 4. Surgical Management of AFX and PDS

Surgery is the most effective approach for treating AFX, with conventional excision and MMS being the two most commonly employed treatment modalities. Due to its ability to deliver a thorough margin analysis, MMS has become increasingly popular for treating AFX. MMS is regarded as more appropriate due to its tissue-sparing resection, which offers cosmetic and reconstructive benefits without increasing the risk of recurrence. This is especially valid for anatomically sensitive areas and immunocompromised patients. A robust systemic review of AFX shows that MMS has a lower recurrence rate (2.0%) than wide local excision (8.7%) [10]. However, other studies have not shown any significant differences in recurrence rates between the two surgical approaches [11]. MMS or conventional excision with a clinical safety margin of at least 0.5 cm is generally recommended for treating AFX [12].

The World Health Organization classification of skin tumors relatively recently recognized PDS as a distinct entity. Due to the scarcity of available data, its optimal management methods are less clear. Further research is required to establish evidence-based recommendations concerning risk stratification and the best management strategies. The main aim of treatment is tumor extirpation while ensuring complete margin control [13]. According to a retrospective study of 92 PDS patients, a safety margin of 2 cm was linked to a lower risk of local recurrence [14]. MMS has also been proposed and utilized for PDS [7,9,15]. In a retrospective study involving 16 cases of PDS, modified MMS achieved a high rate of local disease control (83%) despite a higher recurrence rate after conventional surgery [7]. A broad safety margin, a clinical margin of 2 cm with conventional excision, or MMS is generally recommended for PDS [12]. The safety margin’s extent can be adjusted to the anatomical situation, if necessary.

## 5. Histopathologic Evaluation of Debulk Specimen

The precise staging of tumors requires an evaluation of their high-risk histopathological characteristics. These features may be missed during the initial partial diagnostic skin biopsy due to sampling errors. To mitigate this, formalin-fixed paraffin-embedded permanent sections of the final excision aid in the risk assessment and pathological staging of locally advanced tumors. Appropriate risk assessments and staging are essential for the prognostication and optimization of patient management. Relying only on findings from an initial biopsy may result in under-treatment of high-risk tumors, leading to poor outcomes. 

Mohs micrographic surgery is commonly employed for cutaneous neoplasms in high-risk locations. While MMS allows for a comprehensive evaluation of peripheral and deep margins, the central DS may be discarded, leading to the loss of valuable details about the tumor [16]. To avoid this, Mohs surgeons submit selected central DSs for standard permanent vertical bread-loafed sections of these specimens to be examined by a dermatopathologist. Full-thickness debulking removes the entire visible and palpable tumor with narrow margins. Evaluating the DS via permanent sections is an essential adjuvant of MMS that adds value to the patient’s care. It can help assess aggressive tumor characteristics such as perineural invasion, determine the final pathological tumor stage, and scrutinize unusual frozen section findings, such as a discrepancy between the preoperative biopsy and frozen section diagnosis during MMS.

The evaluation of the central DS by permanent pathology is standard practice for various cutaneous neoplasms, including non-melanoma skin carcinomas like cutaneous SCC, basal cell carcinoma, and Merkel cell carcinoma, melanoma and cutaneous soft tissue tumors such as dermatofibrosarcoma protuberans. For melanomas treated with MMS, analyzing the DS with permanent pathology is essential for accurate staging [17]. In the case of SCC and basal cell carcinoma, a systematic review has shown that tumor debulk analysis helps identify high-risk features [18]. This leads to improved staging accuracy, better treatment decisions, and ultimately better patient outcomes. A study of SCC showed that 19 of 29 (66%) cases were upstaged according to the American Joint Committee on Cancer classification (8th Edition) upon the conventional histopathologic evaluation of MMS debulks [19]. A permanent pathology debulk analysis shows the upstaging of SCC independent of findings on MMS frozen sections. Moreover, the upstaged SCC cases show a more significant likelihood of having large-caliber perineural invasion and are more likely to be referred for adjuvant radiation [20]. 

## 6. Importance of Histopathologic Evaluation of Debulk Specimen of AFX and PDS

Differentiating between AFX and PDS is crucial because PDS tends to have more aggressive clinical behavior [2,5]. For intrinsically high-risk PDS, multidisciplinary management with a comprehensive evaluation utilizing imaging studies is necessary to achieve optimal outcomes. A precise and systematic histopathologic examination is required for the diagnosis of PDS. While the histopathologic assessment of MMS DS is often used for usual non-melanoma skin carcinomas and melanoma, its utility is not commonly discussed regarding AFX and PDS. As the initial diagnostic biopsy is often a shave, the final distinction between AFX and PDS can only be reliably accomplished by histopathologic examination of the entire lesion. 

For AFX treated via MMS, it is prudent to regularly submit DSs for permanent pathology to avoid overlooking PDS cases. While this is especially applicable for large bulky tumors and recurrent lesions, tumors with even superficial subcutaneous infiltration have shown adverse outcomes [4]. This critical step should be included in future guidelines for the surgical treatment of AFX and PDS. A proper distinction will enable appropriate patient workup, the development of accurate staging criteria, and additional treatment modalities [21]. In addition, when the deep extent of the tumor cannot be ascertained due to a superficial initial biopsy, the histopathologic diagnosis of AFX should be made with a caveat. The dermatopathologist should make a cautionary note in the report that the possibility of PDS cannot be excluded as their distinction requires an examination of the entire lesion. Such a comment can alert the Mohs surgeon of the possibility of PDS.

## 7. Conclusions

The clinical, pathological, and treatment comparisons between AFX and PDS are summarized in Table 1. PDS has the potential to be aggressive and metastasize if not controlled locally. PDS should be treated with curative intent via excision with clear surgical margins and subsequent histopathologic examination. The distinction between AFX and PDS requires an examination of the entire lesion. As AFX is commonly treated with MMS, the histopathologic evaluation of its DS by permanent pathology is prudent to avoid the pitfall of underdiagnosing PDS. Future consensus guidelines for AFX and PDS treatment should emphasize the importance of histopathologic evaluation of the DS so that it can become standard practice. Also, when rendering the diagnosis of AFX on superficial biopsies, pathologists should be more proactive in commenting that the possibility of PDS cannot be excluded in partial sampling. These measures can help prevent adverse patient outcomes, such as local recurrence or metastasis, and enable potential additional treatment modalities.

## Figures and Tables

**Table 1:** Clinical, pathological, and treatment comparisons between atypical fibroxanthoma and pleomorphic dermal sarcoma.

	Atypical Fibroxanthoma	Pleomorphic Dermal Sarcoma
**Clinical Presentation**	- Elderly, white, male predominance - Sun-exposed sites, head and neck (ear, scalp) - Localized in dermis - Rapidly growing nodule or plaque, ulceration is common	- Dermal expansile tumor nodule or plaque with involvement of subcutaneous and deeper soft tissue - Rest similar to AFX
**Risk Factor**	- UV exposure - Irradiation - Immunosuppression (e.g., organ transplantation) - Li-Fraumeni syndrome and xeroderma pigmentosum	- Similar to AFX
**Synonym**	- Superficial MFH - Dermal undifferentiated pleomorphic sarcoma	- Superficial MFH - Undifferentiated pleomorphic sarcoma of the skin
**Size**	- Usually between 1 to 2 cm	- Larger than AFX - Median size: 2.5 cm
**Histopathology**	- Highly cellular dermal tumor (Figure 1a,b) - Epithelioid or spindled cells - Striking nuclear pleomorphism and atypia, multinucleated giant cells - Brisk mitotic activity, with atypical forms (Figure 1c,d) - Regression can be seen - Variants include clear cell, granular cell, keloidal, spindle cell, myxoid, sclerotic, pigmented/hemosiderotic, plaque-like	- Infiltration of subcutaneous (Figure 1g) and deeper soft tissue (fascia or muscle) - LVI or PNI in 30% of PDS - Necrosis in 50% of PDS (Figure 1h) - Rest similar to AFX
**Histopathologic Differential Diagnosis**	- Melanoma, poorly differentiated squamous cell carcinoma, angiosarcoma, leiomyosarcoma, dermatofibroma (with monster cells)	- Similar to AFX
**Immunohistochemistry**	- Negative: S100-, SOX10-, melan-A-, cytokeratins-, p40- (Figure 1f), p63-, desmin-, and h-caldesmon- - Positive: CD10+ (Figure 1e), CD68+, CD163+, and smooth muscle actin+	- Similar to AFX
**Genetic Profile**	- Highly mutated tumors - Mutations in *TP53*, *TERT promoter*, *FAT1*, *NOTCH1*, *NOTCH2*, and *CDKN2A*	- *RAS* mutations in 9% of PDS - Rest similar to AFX
**Clinical Behavior**	- Most behave in a low-grade fashion (if strict criteria employed) - 5% local recurrence rate, less than 1% risk of metastasis	- More aggressive clinical behavior - 20–30% local recurrence rate - 10–20% rate of metastasis (skin, lymph node, or lung) - Mortality rate up to 20%
**Surgical Treatment**	- Conventional excision or MMS - Clinical safety margin of at least 0.5 cm - Submit DS from MMS for permanent pathology	- Conventional excision or MMS - Clinical safety margin of 2 cm - Safety margin extent can be adjusted to the anatomical situation - Submit DS from MMS for permanent pathology

AFX, atypical fibroxanthoma; DS, debulk specimen; LVI, lymphovascular invasion; MFH, malignant fibrous histiocytoma; MMS, Mohs micrographic surgery; PNI, perineural invasion; PDS, pleomorphic dermal sarcoma; UV, ultraviolet.

## Data Availability

No new data were created or analyzed in this study. Data sharing is not applicable to this article.

## References

[1] Griewank K.G., Wiesner T., Murali R., Pischler C., Müller H., Koelsche C., Möller I., Franklin C., Cosgarea I., Sucker A. (2018). Atypical fibroxanthoma and pleomorphic dermal sarcoma harbor frequent NOTCH1/2 and FAT1 mutations and similar DNA copy number alteration profiles. Mod. Pathol..

[2] Miller K., Goodlad J.R., Brenn T. (2012). Pleomorphic dermal sarcoma: Adverse histologic features predict aggressive behavior and allow distinction from atypical fibroxanthoma. Am. J. Surg. Pathol..

[3] Iorizzo L.J., Brown M.D. (2011). Atypical fibroxanthoma: A review of the literature. Dermatol. Surg..

[4] van Midden D., Flucke U.E., Amir A.L., Bonenkamp J.J., Lubeek S.F.K., Blokx W.A.M. (2022). Atypical fibroxanthoma and pleomorphic dermal sarcoma: Is superficial infiltration in subcutaneous tissue acceptable in AFX?. Ann. Diagn. Pathol..

[5] Tardío J.C., Pinedo F., Aramburu J.A., Suárez-Massa D., Pampín A., Requena L., Santonja C. (2016). Pleomorphic dermal sarcoma: A more aggressive neoplasm than previously estimated. J. Cutan. Pathol..

[6] Phelan P.S., Rosman I.S., Council M.L. (2019). Atypical Fibroxanthoma: The Washington University Experience. Dermatol. Surg..

[7] Ríos-Viñuela E., Serra-Guillén C., Llombart B., Requena C., Nagore E., Traves V., Guillén C., Vázquez D., Sanmartín O. (2020). Pleomorphic dermal sarcoma: A retrospective study of 16 cases in a dermato-oncology centre and a review of the literature. Eur. J. Dermatol..

[8] López-Llunell C., Yébenes M., Garbayo-Salmons P., Leal L., Mogedas-Vergara A. (2021). Atypical fibroxanthoma relapse as pleomorphic dermal sarcoma after slow Mohs micrographic surgery. Int. J. Dermatol..

[9] Kim J.I., Choi Y.J., Seo H.M., Kim H.S., Lim J.Y., Kim D.H., Chae S.W., Lee G.Y., Kim W.S. (2016). Case of Pleomorphic Dermal Sarcoma of the Eyelid Treated with Micrographic Surgery and Secondary Intention Healing. Ann. Dermatol..

[10] Tolkachjov S.N., Kelley B.F., Alahdab F., Erwin P.J., Brewer J.D. (2018). Atypical fibroxanthoma: Systematic review and meta-analysis of treatment with Mohs micrographic surgery or excision. J. Am. Acad. Dermatol..

[11] Orholt M., Aaberg F.L., Abebe K., Walsh S., Roenigk R.K., Venzo A., Schmidt G., Klyver H., Jensen D.H., Herly M. (2022). Risk factors for local atypical fibroxanthoma recurrence and progression to pleomorphic dermal sarcoma: A meta-analysis of individualized participant data. J. Surg. Oncol..

[12] Helbig D., Ziemer M., Dippel E., Erdmann M., Hillen U., Leiter U., Mentzel T., Osterhoff G., Ugurel S., Utikal J. (2022). S1-guideline atypical fibroxanthoma (AFX) and pleomorphic dermal sarcoma (PDS). J. Dtsch. Dermatol. Ges..

[13] Lonie S., Yau B., Henderson M., Gyorki D., Angel C., Webb A. (2020). Management of pleomorphic dermal sarcoma. ANZ J. Surg..

[14] Persa O.D., Loquai C., Wobser M., Baltaci M., Dengler S., Kreuter A., Volz A., Laimer M., Emberger M., Doerler M. (2019). Extended surgical safety margins and ulceration are associated with an improved prognosis in pleomorphic dermal sarcomas. J. Eur. Acad. Dermatol. Venereol..

[15] Kurtti A., Farhadian J., Meehan S., Madu P., Bradu S. (2022). A case of pleomorphic dermal sarcoma with perineural invasion treated with Mohs micrographic surgery and adjuvant radiation therapy. Dermatol. Online J..

[16] Geisse J.K. (2000). Mohs surgery: Debulking specimens-to submit or discard-that is the question. Dermatol. Surg..

[17] Iorizzo L.J., Chocron I., Lumbang W., Stasko T. (2013). Importance of vertical pathology of debulking specimens during Mohs micrographic surgery for lentigo maligna and melanoma in situ. Dermatol. Surg..

[18] Singh B., Dorelles A., Konnikov N., Nguyen B.M. (2017). Detection of High-Risk Histologic Features and Tumor Upstaging of Nonmelanoma Skin Cancers on Debulk Analysis: A Quantitative Systematic Review. Dermatol. Surg..

[19] Weinhammer A., Bennett D.D., Eickhoff J., Xu Y.G. (2022). Histopathologic Evaluation of Cutaneous Squamous Cell Carcinoma Debulk Specimens Before Mohs Micrographic Surgery and Its Influence on Squamous Cell Carcinoma Staging. Dermatol. Surg..

[20] Nemeh M.N., Srivastava D., Nijhawan R.I. (2022). Value of permanent pathology for debulk and Mohs specimens during Mohs micrographic surgery for cutaneous squamous cell carcinoma: A retrospective cohort study. J. Am. Acad. Dermatol..

[21] Helbig D., Klein S. (2022). Immune checkpoint inhibitors for unresectable or metastatic pleomorphic dermal sarcomas. Front. Oncol..

